# Designing Lubricating Properties of Vegetable Base Oils

**DOI:** 10.3390/molecules23082025

**Published:** 2018-08-14

**Authors:** Jolanta Iłowska, Justyna Chrobak, Rafał Grabowski, Michał Szmatoła, Julia Woch, Iwona Szwach, Jolanta Drabik, Magdalena Trzos, Rafał Kozdrach, Małgorzata Wrona

**Affiliations:** 1 Institute of Heavy Organic Synthesis “Blachownia”, Energetykow 9, 47-225 Kedzierzyn-Kozle, Poland; ilowska.j@icso.com.pl (J.I.); grabowski.r@icso.com.pl (R.G.); szmatola.m@icso.com.pl (M.S.); woch.j@icso.com.pl (J.W.); szwach.i@icso.com.pl (I.S.); 2 The Institute for Sustainable Technologies—National Research Institute, Kazimierza Pulaskiego 6/10, 26-600 Radom, Poland; jolanta.drabik@itee.radom.pl (J.D.); magdalena.trzos@itee.radom.pl (M.T.); rafal.kozdrach@itee.radom.pl (R.K.); malgorzata.wrona@itee.radom.pl (M.W.)

**Keywords:** design of experiments, lubricant base oil, lubricating properties, vegetable oil, *Crambe abyssinica* oil, chemical modification, oxidation, *N*-hydroxyphthalimide

## Abstract

Lubricants that are based on renewable raw materials have drawn increased attention in various applications, especially those related to the food industry. Due to the high requirements of environmental protection, there is a need to develop biodegradable base oils that are environmentally friendly and do not contain harmful components. The objective of the research was to obtain a base oil with a certain viscosity and certain desired lubricating properties. Base oils were obtained from *Crambe abyssinica* oil by means of blending with synthetic oil and oxidation. The oxidation processes were carried out in the presence of *N*-hydroxyphthalimide with or without CO_2_ as a solvent. As a final result of this study, oil bases meeting the viscosity requirements and showing suitable lubricating properties were obtained. The Raman spectra of the obtained oils were evaluated.

## 1. Introduction

Nowadays, mostly mineral and synthetic lubricants are available in the markets of Poland and Europe. In the last few years, about 75% of the world production of base oils were conventional mineral oils, which are obtained from petroleum [1]. According to the American Petroleum Institute (API) classification, such oils belong to Group I of base oils and are evaluated as products with low biodegradability. Groups II and III consist of unconventional mineral oils, which are obtained from petroleum but have enhanced composition. Their share in the market amounts to 15% of the yearly world production. Polyalphaolefin oils and the other base oils that belong to Groups IV and V constitute the remaining 10% of production [2]. Due to the necessity to reduce the harmful impact of lubricants on the environment, there has been a growing demand for ecological, biodegradable oils.

Unmodified vegetable oils are used as base oils only for applications with a low thermal load due to their limited thermal and oxidative stability [3]. Vegetable oils that are used in lubricating products mostly have modified structures, which are obtained through the genetic modification of crops or through chemical transformations [4,5]. The chemical modification that has been already described in the literature may be accomplished using processes, such as selective hydrogenation, transesterification, epoxidation, hydroformylation, alkylation, Friedl–Crafts acylation, or metathesis [6].

Technology improvements that are related to non-toxic components require the use of vegetable oils as a dispersing phase. Such oils have different viscosity classes that can be obtained through modification processes or by mixing vegetable oil with synthetic oils with a high viscosity class [7]. The modification of vegetable oil for obtaining oils with different viscosity classes has already been achieved and is the subject of our patent applications [8,9]. The modification process involves the oxidation of the vegetable oil under certain conditions. 

The high temperature used during the process has several positive effects, such as an increase in stability and viscosity of the oil base. However, this high temperature also causes disadvantageous degradation of vegetable oils. Lowering the process temperature is possible with the use of oxidation reaction catalysts, such as *N*-hydroxyphthalimide [10], and the addition of easily removable solvent, which may be the supercritical carbon dioxide (scCO_2_).

**Figure 1:**
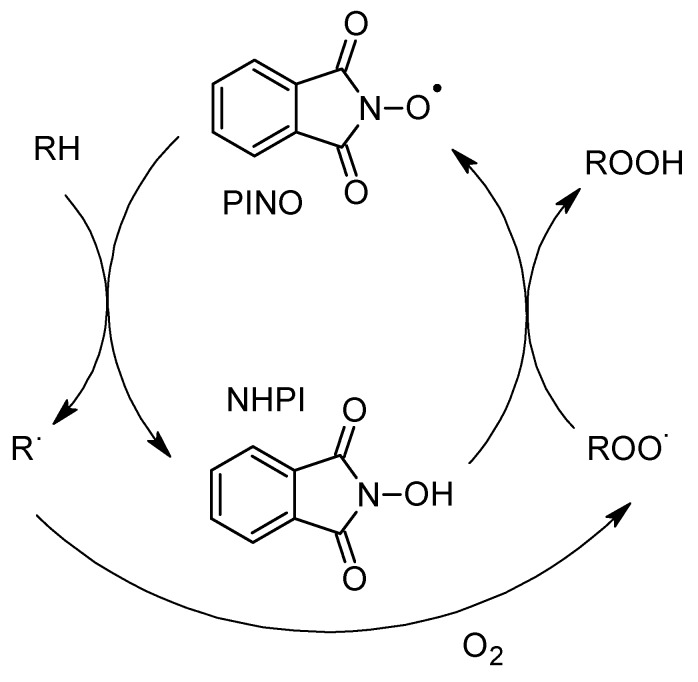
The principle of *N*-hydroxyphtalimide (NHPI) catalytic cycle.

For the modification of vegetable oils, *N*-hydroxyphtalimide (NHPI) was used as a catalyst for oxidation. The catalytic cycle starts with the creation of the phtalimide-*N*-oxyl (PINO) radical in the reaction of NHPI with peroxyl radicals [11]. The derived radical reacts with hydrocarbon, leading to NHPI regeneration (Figure 1). According to the reports in the literature, NHPI catalytic activity in the modification of vegetable oils has not yet been tested.

ScCO_2_ has many advantages as a solvent: it is easily available, cheap, safe for the environment, non-toxic, and inert in the oxidation processes. Furthermore, it excellently blends with oxygen and other gases, has a high diffusion coefficient and low viscosity, and has a high thermal conductivity. Finally, it can be easily separated from the reaction mixture [12]. The application of scCO_2_ enables us to lower the degeneration grade of vegetable oils to unwanted low molecular compounds.

The object of the present research was to obtain vegetable base oils from *Crambe abyssinica* (crambe, Abyssinian kale) oil, which is characterized by a high content of erucic acid and natural antioxidants [13]. Abyssinian oil is more commonly known as Crambe oil, which is a part of the mustard family of plants. The use of non-edible oils in the lubricants industry is very important because of the immense demand for edible oils as a food source. Mainly for this reason, the Abyssinian oil was selected as a raw material for our research. The vegetable oil was modified to obtain a higher viscosity class oil (VG 150) by blending it with synthetic oil and through the process of chemical modification, which was conducted in diversified conditions in accordance with Taguchi’s experiment plan.

The physicochemical and lubricating properties of obtained oils were evaluated. Based on this evaluation, the effect of modification on determining the rheological and lubricating properties was characterized. These oils can be applied as lubricating agents, including their application as lubricating oils (with the enriching additives) or as the dispersive phase of plastic greases [7].

## 2. Results and Discussion

### 2.1. Abyssinian Oil Modification

Within the framework of the research, the modification of vegetable oil from *Crambe abyssinica* involved its oxidation in the presence of the catalyst and was carried out in the presence of supercritical carbon dioxide (as a solvent) in a pressure reactor according to the experimental plan (Table 1 and Table 2). The research plan was prepared with the use of the Design of Experiments methodology (DOE, see Section 3.1).

All modified oils after the experiments were tested to determine kinematic viscosity, peroxide number (LN), iodine number (LJ), saponification number (LZ), and acid number (LK).

### 2.2. Statistical Analysis of Optimization Experiments

The results of the conducted experiments were analyzed. The Eta function value (meaning the ratio of signal factors to noise factors) for individual values of dependent variables was determined. The maximal values of the Eta function show the best values of process parameters, considering the assumed criteria.

**Figure 2:**
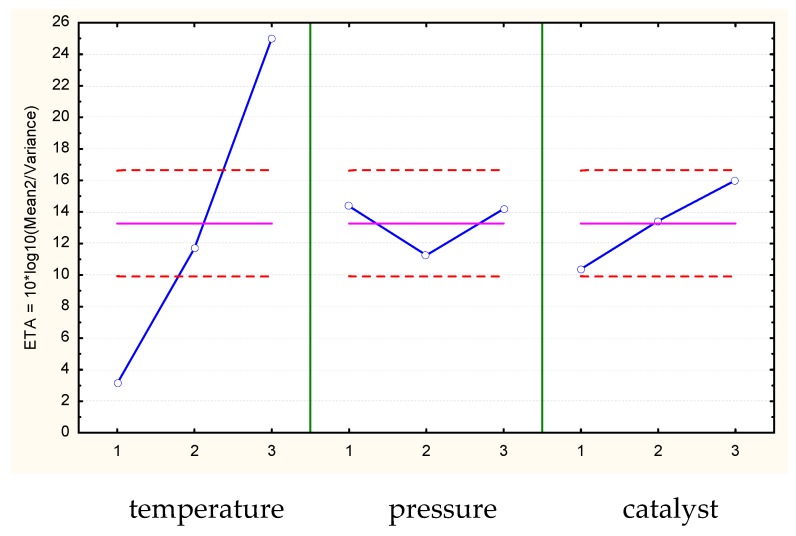
Average values of the Eta function (blue line - meaning the ratio of signal factors to noise factors) for individual parameter values (temperature, pressure, and catalyst) of oil modification with the use of CO_2_ solvent (Abyssinian oil, nominal viscosity of 150 mm^2^/s). Mean value is equal to 13.273 (pink). Dashed line indicates ±2 × standard error (red).

**Figure 3:**
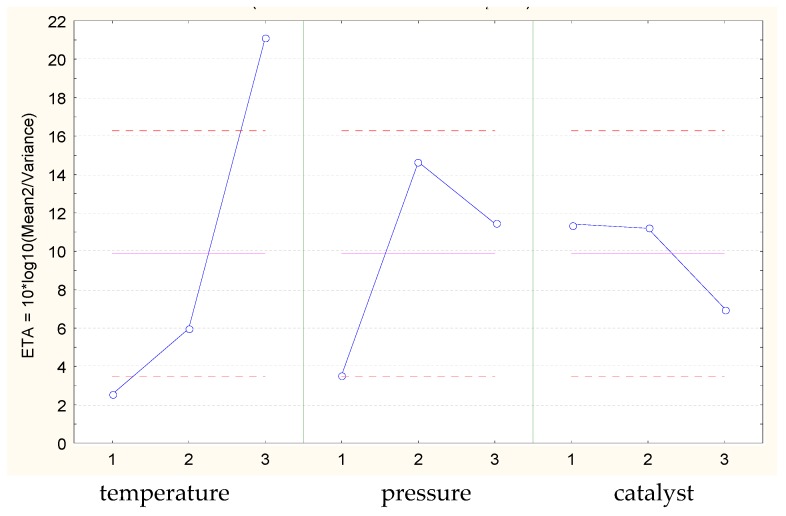
Average values of the Eta function (blue) for individual parameter values (temperature, pressure, and catalyst) of oil modification without the use of CO_2_ (Abyssinian oil, nominal viscosity of 150 mm^2^/s). Mean value is equal to 9.882 (pink). Dashed line indicates ±2 × standard error (red).

Figure 2 shows the Eta function values for the oil modification processes with use of CO_2_, and Figure 3 shows the values for the processes without CO_2_.

In Table 3, the ANOVA (variation analysis) results of the analyzed oil modification process experiments were presented.

When a catalyst is included in the processes without CO_2_, the confidence level is significantly lower than the assumed 95% confidence interval. We also did not reach the assumed level of statistical significance in the processes with CO_2_ pressure parameters, although there was only a small increase above this level.

The shares of the individual parameters in the variance are shown in the table and also shown through the Eta function values in Figure 2 and Figure 3. This shows that the process temperature significantly influences the determination of the assumed value of the modified oil viscosity independently of CO_2_. The other parameters of the modification process have a smaller influence. However, as the variance analysis implies, even although other parameters have a lower impact than the temperature in the explanation of dependent variable variability, all the process parameters have an impact on obtaining the desired value of oil viscosity. In the case of processes that use CO_2_, there is a model error called rest (Table 3), which represents the unexplained (with use of considered parameters) variation of output quantity that has a lower impact.

### 2.3. Model Verification

The verification of prognoses was carried out. Due to the statistical analysis of optimization experiments, the optimal parameters for obtaining products with a viscosity class of VG 150 and projected viscosity values were obtained (Table 4).

The processes of Abyssinian oil oxidation according to obtained parameters were carried out. After this, the acquired A_O_2_ and A_CO_2_ modified oils were analyzed (Table 5).

To summarize, the values of projected oil viscosities and experimentally determined values were as follows:

- A_CO_2_ oil (process with the use of solvent): the projected viscosity value was 150.7 ± 14.6 mm^2^/s. The value obtained in the experiment was 143.4 mm^2^/s.
- A_O_2_ oil (process without use of solvent): the projected viscosity value was 150.6 ± 13.1 mm^2^/s. The value obtained in the experiment was 156.9 mm^2^/s.

Based on the verification tests carried out, the high quality of prognoses was confirmed. The prognosis error, which was specified as the absolute error calculated on the basis of the quotient of the prognosis difference and the experimental value compared with the experimental value, was calculated for the processes without the use of solvent as 5% and for the processes with the use of solvent as 4%.

The values of modified oil viscosity, which were obtained in the process with the parameters that were assumed on the basis of the prognosis ensuring the acquisition of the assumed accuracy class, had satisfactory precision in the range of prognosis tolerance and in the range of viscosity determined by the assumed VG 150 class.

### 2.4. Properties Evaluation

The characteristics of the rheological properties of the oils obtained as a result of the blending (Abyssinian oil A with synthetic oil S in S/A 1:1.5 ratio and S/A 1:1.7 ratio, *w*/*w*) and the modification in comparison to the initial oil A are displayed in Table 6.

Based on the obtained results, we stated that the oils after the modification were characterized by the diversified viscosity–temperature properties.

#### 2.4.1. Characteristics of Lubricating and Rheological Properties

**Figure 4:**
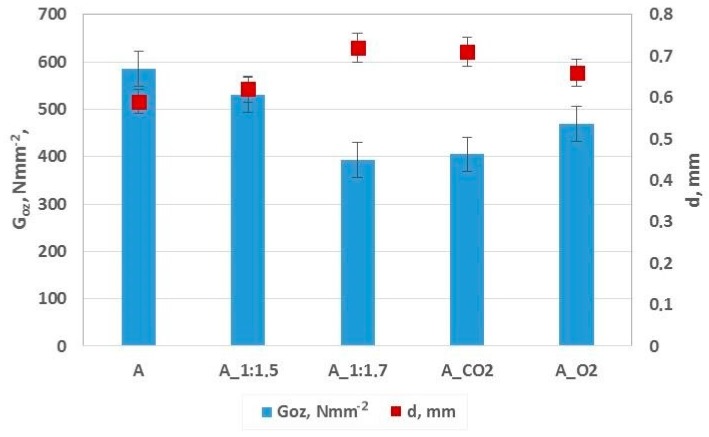
Lubricating characteristics of Abyssinian oil A and oils obtained through blending A_1:1.5 and A_1:1.7 and also A_CO_2_ and A_O_2_ in the modification process with a Goz/40 limiting wear load.

The anti-wear properties of the base oils were determined based on the 1-h test with a node load of 392 N and a spindle rotation of 500 ± 20 turn/min in the temperature of 20 ± 5 °C. After the 1-h wear test, the traces of wear on the surface of the immobile balls were evaluated. The measurements of the diameter of the ball wear traces were conducted with the use of an optical microscope. The size of the wear traces in the directions that are parallel and perpendicular to the direction of friction were measured. G_OZ_/40 value was calculated from the correlation, in which the average diameter of the wear trace and load of the friction node was taken into account (Figure 4).

The oils obtained through modification (A_CO_2_ and A_O_2_) were characterized by anti-wear properties that are comparable with the oils of the same viscosity class obtained through the blending with synthetic oil. A_1:1.7 was similar to A_CO_2_ and A_1:1.5 was similar to A_O_2_.

**Figure 5:**
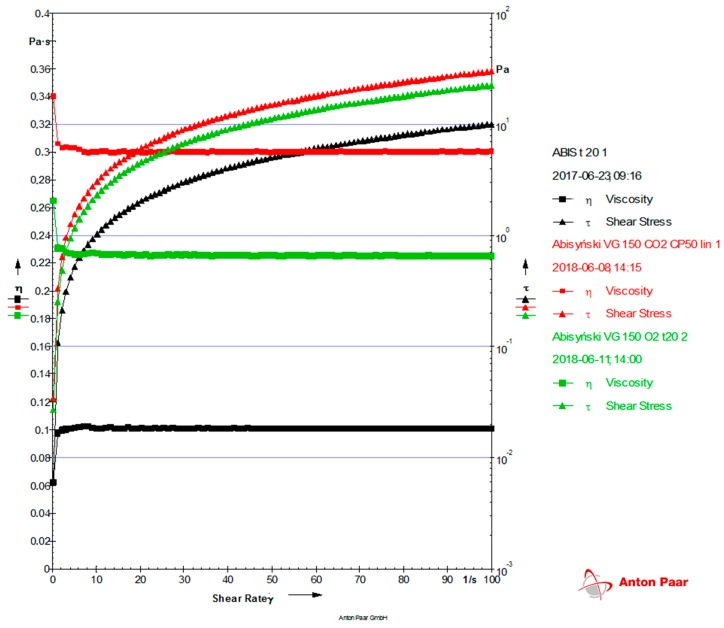
Flow and viscosity curves at a temperature of 20 °C for Abyssinian oil A (black) and the oils obtained through modification—A_CO_2_ (red) and A_O_2_ (green).

For the determination of the rheological properties of the base oils obtained through modification, a MCR-101 classic rotary rheometer with air bearing was used. The flow and viscosity curves were determined at 20 °C. The measurements were implemented in the range of shear velocity of 0.1–100 s^−1^ with a constant temperature. The viscosity and flow curves for A_O_2_ and A_CO_2_ modified oils were additionally compared with an initial vegetable oil A (Figure 5). Comparing the properties of the evaluated oils in the range of shear velocity, we found that oil A is characterized by significantly lower viscosity values in comparison with the A_O_2_ and A_CO_2_ oils.

The flow curves show the dependence of viscosity on shear velocity and are constant in the case of Newtonian fluids for a particular medium in a particular temperature. The viscosity curves are equivalent to the flow curves (Figure 5).

As a result of the modification, oil bases with a higher viscosity were obtained, which were classified to the VG 150 viscosity class. This is positive considering the planned application, as it is compatible with the project assumptions [7].

#### 2.4.2. Raman Spectroscopy

**Figure 6:**
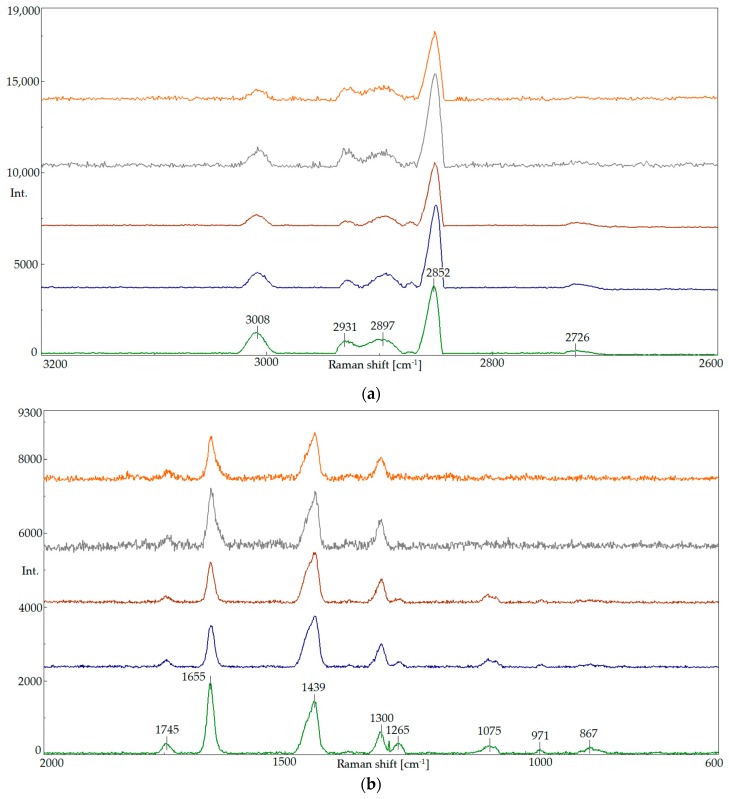
Raman spectra of Abyssinian oil A (green); oils obtained through blending, which are namely A_1:1.5 (blue) and A_1:1.7 (brown); and oils after the modification process, which are namely A_CO_2_ (grey) and A_O_2_ (orange), in the range of the Raman frequency shift: (**a**) 3200 cm^−1^–2600 cm^−1^ and (**b**) 2000 cm^−1^–600 cm^−1^.

The chemical structure of oils was evaluated with the use of Raman spectroscopy, which was applied to identify the changes in the vegetable oil quality that are caused by the modification process. Raman spectroscopy allows us to determine the unsaturated ratio of polyunsaturated fatty acids and the presence of C=C bond trans and cis isomers. For this reason, it was used to evaluate the effect of the modification process on the change in the chemical structure of oils. Spectra were obtained for the initial Abyssinian oil, the oils obtained through blending (A_1:1.5 and A_1:1.7), and oils after the modification process (A_CO_2_ and A_O_2_) (Figure 6).

The spectra of A/S oil mixtures had the same bands as the vegetable oil A spectra, but the intensity of those bands was changed. The intensity of the band at 1654 cm^−1^ band was generally reduced. For the A_1:1.5 mixed oil, there was a growth of the band at 2850 cm^−1^ that could be observed in the comparison with vegetable oil A. For the A_1:1.7 mixture, the bands at the frequency of 3100–2800 cm^−1^ shrunk. The unsaturated ratio of fatty acids that was determined on the basis of the I1263/I1300 intensity ratio was lower compared with vegetable oil A, which was the lowest for the A_1:1.7 oil.

In the spectra of oils obtained through modification (A_CO_2_ and A_O_2_), we observed that the use of carbon dioxide caused an increase in the intensity of the bands at 2850 and 2931 cm^−1^ in the A_CO_2_ spectrum, while there was a decrease in the band intensity at 3008 cm^−1^. On the other hand, under the influence of oxygen, the intensity of the bands in the A_O_2_ oil was lowered at 3008 and 1655 cm^−1^. Furthermore, both processes caused lowering in the unsaturated ratio of fatty acids. In the spectra of the modified oil, the band at 1263 cm^−1^ almost disappeared. The band at 1670 cm^−1^ that is typical for undesired trans isomers was not present in the spectra. This information is rather advantageous because it confirms that the modification process does not cause the formation of harmful oxidation products.

## 3. Materials and Methods

### 3.1. Design of Experiments

The aim of the conducted research was to obtain VG 150 viscosity class oil and because of that, the viscosity range of 135–165 mm^2^/s was targeted. It was also assumed that the modified oil should reach a viscosity that is as close as possible to 150 mm^2^/s. To determine the oil modification process parameters to result in an oil with the required viscosity, an optimization experiment was planned. The experiment plan was developed in accordance with Taguchi’s approach, which assumes the “best nominal” criteria [14,15]. Dependent variables were assumed: temperature, pressure, and catalyst content in accordance with the technological parameters of the oil modification process. Each independent variable could take one of three specified values. The changes in viscosity were analyzed, while the nominal variable was assumed to be equal to 150 mm^2^/s. The experimental plan was composed of 9 systems according to the orthogonal array.

According to the experimental plan, we trialed two different processes without the presence of carbon dioxide (Table 1) and with use of carbon dioxide (Table 2).

### 3.2. Method of Oxidation

The oxidation processes of Abyssinian oil were conducted using equipment that was composed of three devices: a Buchi “Limbo” pressure reactor (Uster, Switzerland), a Blue Shadow metering pump (Berlin, Germany), and a Julabo thermostat (Seelbach, Germany). The processes were carried out according to the experimental plan (the conditions in Table 1 and Table 2). The raw materials (oil and a specified amount of catalyst) were placed into the pressure reactor together with oxygen and carbon dioxide. The pressure was controlled during the process. An oxidation process initiator azobisisobutyronitrile (AIBN) was used in the amount of 0.05%. During the modification process, Abyssinian oil from ZielonyKlub company (Kielce, Poland) was used, which is characterized by the following physicochemical properties: 0.906–0.911 g/cm^3^ density; 48.20 cSt kinematic viscosity at 40 °C; 2.80 meq O_2_/kg peroxide number; 83.48 g I_2_/100 g iodine number; 170.50 mg KOH/g saponification number; and 0.34 mg KOH/g acid number. 

### 3.3. Determination of Modified Oils Properties

Viscosity–temperature oil properties were evaluated based on the kinematic viscosity determined at the temperature of 40 °C and 100 °C in accordance with PN EN ISO 3104:2004 norm and the viscosity index in accordance with PN-ISO 2909:2009 norm. The obtained modified oils were tested through the determination of kinematic viscosity; the peroxide number in accordance with PN-EN ISO3960:1996 norm; the iodine number in accordance with PN-87/C-04281 norm; the saponification number in accordance with PN-C-04288-07:1988 norm; and the acid number in accordance with PN-ISO 660:1998 norm.

### 3.4. Evaluation of Lubricating and Rheological Properties

The lubricating properties of oils were characterized on the basis of a 1-h-long anti-wear test with the use of a four-ball tester (T-02). As a criterion for the lubricating efficiency of the tested oils, a reduction in the wear trace diameter (the increase of limiting wear load) was assumed [16].

For the determination of the rheological properties of the oils, a MCR-101 Anton Paar rotary rheometer (Graz, Austria) with air bearing was used. The rheometer control and measurement data analysis were carried out using Rheoplus software. The measurements were conducted using a cone-plate (CP50-1) measurement system. The viscosity and flow curves were determined.

### 3.5. Raman Spectroscopy

The Raman spectra [17] were obtained using a NRS 5100 Jasco confocal grating Raman microspectrometer (Tokyo, Japan), which was equipped with a pumped laser with a wave length of 532 nm and CCD (Charge Coupled Device) detector. The operating conditions of the spectrometer were as follows: diffraction grating of 1800 lines/mm; laser power of 5.1 mW; numerical aperture of 4000 μm; resolution of 8.4 cm^−1^; lens of magnification of 20×, and exposure time of 15 s.

## 4. Conclusions

The method for the preparation of modified vegetable oils is the result of research that will enable the use of the obtained oils as a base in environmentally friendly lubricants. 

During the creation of specialized lubricating agents, particular attention was paid to choosing components, taking into consideration both their functional properties and their ecological properties.

The effect of the modification and blending with synthetic oil is a product with different properties compared with the properties of the crude Abyssinian oil. The choice of components and the modification process conditions ensured that during the modification process, high-quality oil bases with complex viscosity–temperature properties and advantageous lubricating properties would be obtained.

In the Raman spectra of the oils, no undesired isomers were detected.

## 5. Patents

The research described above is the subject of two patent applications concerning vegetable oils modification: application No. P. 425863 and No. P.425873.

## Figures and Tables

**Table 1:** Plan for the experiment of vegetable oil modification with oxygen.

System No.	Temperature, °C	O_2_ Pressure, MPa	Catalyst, %
1	80	0.1	0
2	80	0.4	0.05
3	80	0.6	0.5
4	100	0.4	0.5
5	100	0.6	0
6	100	0.1	0.05
7	120	0.6	0.05
8	120	0.1	0.5
9	120	0.4	0

**Table 2:** Plan for the experiment of vegetable oil modification with oxygen in the presence of carbon dioxide.

System No.	Temperature, °C	Σ Pressure, MPa	Catalyst, %
1	80	10	0
2	80	15	0.05
3	80	20	0.5
4	100	10	0.5
5	100	15	0
6	100	20	0.05
7	120	10	0.05
8	120	15	0.5
9	120	20	0

**Table 3:** Results of variation analysis for models with and without solvent.

	Processes without CO_2_				Processes with CO_2_			
	SS	df	*p*	% Share	SS	df	*p*	% Share
Temperature	1760.55	2	0.000	58.3%	2190.32	2	0.000	85.7%
Pressure	585.21	2	0.001	19.4%	54.96	2	0.059	2.1%
Catalyst	109.90	2	0.169	3.6%	143.07	2	0.002	5.6%
Rest	565.24	20		18.7%	168.36	20		6.6%
SUM	3020.90			100.0%	2556.71			100.0%

SS: sum of squared deviations from the mean–variance function, df: degrees of freedom, P: significance level measure (*p* ≤ 0.05 for confidence level ≥95%), and % share: percentage share of parameter in the explanation of output quantity variation.

**Table 4:** Data obtained from the model; projected viscosity and conditions for the Abyssinian oil modification process with CO_2_ (A_CO_2_) or without CO_2_ (A_O_2_)_._

Oil	A_O_2_	A_CO_2_
Temperature, °C	120	120
Pressure O_2_/Σ, MPa	0.4	10
Catalyst, %	0.05	0.05
Projected viscosity in 40 °C, cSt	150.6	150.7

**Table 5:** Results of the experiments confirming the correctness of the mathematical model for the Abyssinian oil modification.

Oil	A_O_2_	A_CO_2_
Viscosity 40 °C, cSt	156.9	143.4
LN, meq O_2_/kg	15.16	13.10
LJ, g I_2_/100 g	54.10	63.70
LZ, mg KOH/g	214.23	248.70
LK, mg KOH/g	25.70	28.05

LN, peroxide number; LJ, iodine number; LZ, saponification number; and LK, acid number.

**Table 6:** Viscosity–temperature characteristics of modified vegetable oil A.

Parameters	Determination Method (Standard Number)	Tested Oils				
		Oil	Blending		Modification	
		A	A_1:1.5 S/A 1:1.5	A_1:1.7 S/A 1:1.7	A_CO_2_	A_O_2_
Kinematic viscosity in temp. of 40 °C, mm^2^/s	PN EN ISO 3104:2004	46.9	140.2	171.4	143.4	156.9
Kinematic viscosity in temp. of 100 °C, mm^2^/s	PN EN ISO 3104:2004	10.1	22.8	24.4	16.1	17.2
Viscosity indicator, WL	PN ISO 2909:2009	208	192	184	138	138
VG viscosity class according to ISO	ISO 3448	46	150	150/220	150	150
